# Comparison of hepatitis E virus seroprevalence between HBsAg-positive population and healthy controls in Shandong province, China

**DOI:** 10.1186/s12879-018-2974-3

**Published:** 2018-02-12

**Authors:** Li Zhang, Zechun Jiang, Jingjing Lv, Jiaye Liu, Bingyu Yan, Yi Feng, Li Li, Guomin Zhang, Fuzhen Wang, Aiqiang Xu

**Affiliations:** 1 Academy of Preventive Medicine, Shandong University, Jinan, China; 2 Shandong Provincial Key Laboratory of Infectious Disease Control and Prevention, Shandong Center for Disease Control and Prevention, Jinan, China; 3 Rushan Center for Disease Control and Prevention, Weihai, China; 4 Chinese Center for Disease Control and Prevention, Beijing, China

**Keywords:** Hepatitis E virus, Chronic hepatitis B, Seroprevlance, Superinfection

## Abstract

**Background:**

Persons with chronic hepatitis B (CHB) infection were reported to suffer severe disease after hepatitis E virus (HEV) superinfection, but the studies regarding HEV seroprevalence in this population were limited. A recent study in Vietnam found higher HEV seroprevalence among CHB patients compared with healthy controls.

**Methods:**

A community-based case-control study was conducted in two counties of Shandong province, China, where hepatitis E incidence was at the highest (Rushan) and lowest (Zhangqiu) in the province based on data from routine public health surveillance. Four townships were selected randomly from each county and all residents in these townships were tested for hepatitis B surface antigen (HBsAg). Those tested positive for HBsAg (CHB group) and the 1:1 age and sex-matched HBsAg-negative residents (control group) were included. Anti-HEV IgM and IgG were tested and positive rates of IgG and IgM were compared between the CHB group and the control group.

**Results:**

In total, 2048 CHB participants and 2054 controls were included in the study. In the CHB group, HEV IgG seroprevalence was 9.16% (95% *CI*: 7.47–11.09) in Zhangqiue and 38.06% (95% *CI*: 35.07–41.19) in Rushan (*P* < 0.001); the corresponding rates of IgM were 0.1% (95% *CI*: 0.002–0.54) and 1.57% (95% *CI*: 0.90–2.53), respectively (*P* < 0.001). HEV IgG seroprevalence was similar between CHB group and the control group in both counties (*P* = 0.21, *P* = 0.47, respectively) and the same results were found for the positive rate of IgM (*P* = 0.103, *P* = 0.262, respectively). Multivariable analysis showed the status of HBsAg was not independently associated with the status of anti-HEV IgG in either Zhangqiu or Rushan [*P* = 0.187, *OR* = 1.23(95% *CI*: 0.90, 1.68); *P* = 0.609, *OR* = 1.05 (95% *CI*: 0.87, 1.26)].

**Conclusions:**

The seroprevalence of HEV varies greatly in different geographic areas, but the seroprevalence is similar between populations with and without CHB. CHB patients residing in high HEV endemic areas might be at higher risk for HBV-HEV superinfection.

## Background

Hepatitis E virus (HEV) was discovered in 1980’s and has been documented to be prevalent in many countries [1]. HEV seroprevalence among the general population was 1.95% in the Netherlands [2], 5.3% in Japan [3] and 21% in the United States [4]. HEV is among the leading causes of acute viral hepatitis in developing countries [5] and is responsible for approximately 56,600 deaths in the world annually [6]. HEV infection is often asymptomatic, however in some special populations including pregnant women, patients with chronic hepatitis and those who are immunosuppressed, it might cause severe disease or chronic infection [7–9].

Although HEV is primarily transmitted by the faecal-oral route, its transmission by transfusion has already been documented [5]. HEV RNA was detected in 0.001% to 0.33% of blood donors in Australian, the United States and Qatar [10–12], suggesting the risk of HEV infection through transfusion. It is well known that hepatitis B and C could also be transmitted by transfusion. The studies from Turkey and Sweden found the seroprevalence of HEV was significantly higher among chronic hepatitis C patients compared with the general population [13, 14]. A study in Vietnam found significantly higher seroprevalence of HEV among chronic hepatitis B (CHB) patients [15], while the study in France found no difference [16].

Both hepatitis B and hepatitis E are endemic in China. The seroprevalence of hepatitis B surface antigen (HBsAg) and anti-HEV IgG was 7.18% and 23.1%, respectively according to a national survey in 2006 [17, 18]. The estimated number of persons with CHB is up to 90 million in China [17]. Although the superinfection of HBV and HEV has been widely reported in China [19, 20], all available studies are hospital-based focusing on clinical outcomes after superinfection. HEV seroprevalence among persons with CHB is poorly understood in China.

We conducted this study to evaluate HEV seroprevalence among persons with and without CHB in Shandong province, China.

## Methods

### Study population

**Fig. 1:**
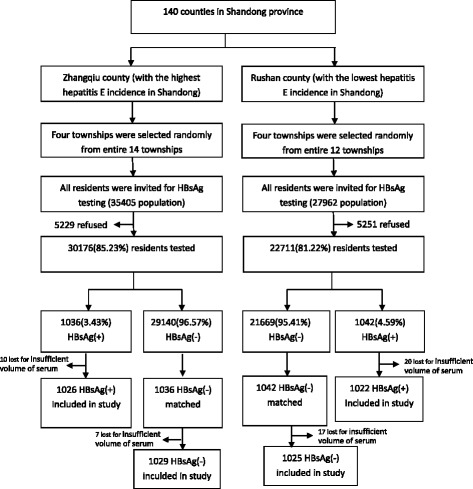
Study participant flow chart

Shandong province is located in eastern China. The province has 140 counties and a population of 97 million. The average seroprevalence of anti-HEV IgG was 11% among the general population [21]. This study was conducted during April and July 2014. The study population was selected by two-stage sampling method. First, all counties were ranked by HEV incidence reported through China National Notifiable Disease Reporting System (NNDRS) in 2013 and the county with the highest (Rushan) and the lowest (Zhangqiu) HEV incidence were selected. Second, four townships were selected by simple random sampling from each county. All residents in these townships were tested for HBsAg. All HBsAg-positive inhabitants and 1:1 age and sex-matched inhabitants negative for HBsAg were included in the study. The study flow chart is shown in Fig. 1.

### Questionnaire survey

A face-to-face interview was conducted by the staff from the county level Center for Disease Control and Prevention (CDC). The information was collected on age, gender, education attainment, special occupations (seafood cultivation, processing and selling; swine laughter or selling), health habits including washing hands before dining, drinking boiled water and not-eating out of home and the histories of chronic diseases such as hypertension, heart disease, stroke, etc.

### Sample collection and testing

Blood samples of 5 ml were collected from each participant. HBsAg was detected using Xinchuang ELISA kits (Xinchuang Biology Co., Xiamen, China). Anti-HEV IgG and IgM were detected using the Wantai ELISA kits (Wantai Biology Co., Beijing, China). HEV RNA was tested by real-time PCR for the serum positive for anti-HEV IgM (Invitrogen, one step qRT-PCR system). All tests were performed following the manufacturers’ instructions and were conducted by staff at Shandong Provincial CDC.

### Medical examination for HBsAg-positive participants

Further medical examination was carried out for HBsAg-positive participants in local hospitals, including physical examination, Ultrasound examination of liver, testing of HBV serological markers including antibody against HBsAg (anti-HBs), antibody against hepatitis B core antigen (anti-HBc), hepatitis B e antigen (HBeAg) and antibody against hepatitis B e antigen (anti-HBe), and HBV DNA level and Alanine aminotransferase (ALT) level. The participants were classified into HBV carrier, CHB patients, cirrhosis and hepatocellular carcinoma (HCC) according to the above medical examination. HBV carrier was defined as: (1) Tested positive for HBsAg; (2) had no signs and symptoms suggestive of hepatitis; (3) ALT level was within normal limit (< 40 IU/ml) [22]; (4) B ultrasound examination did not find any abnormalities in the liver. A case of chronic hepatitis patient was defined as: (1) HBsAg seropositive status lasted for 6 months or beyond; (2) had signs and symptoms suggestive of hepatitis; (3) HBV DNA was positive and ALT level increased(≥40 IU/ml) and (4) B ultrasound examination showed chronic liver disease. Cirrhosis and HCC were diagnosed mainly according to ultrasound findings.

### Statistical analyses

The seroprevalence of anti-HEV IgG and IgM across different groups was assessed with Pearson Chi-square test, trend Chi-square test and fisher’s exact test as appropriate. Multivariable logistic regression model was built to estimate the independent association between status of anti-HEV IgG and HBsAg. The analyses were conducted with STATA 10.0 and the *P* value < 0.05 was considered to be statistically significant.

### Ethical issues

This study was approved by Ethics Committee of Shandong Provincial CDC and a written informed consent form was signed by each participant.

## Results

### Demographic characteristics of the participants

As shown in Tables 1, 1026 HBsAg-positive participants (age: 51.13 ± 13.42 years, age range: 6–85 years) and 1029 HBsAg-negative participants (51.62 ± 13.40 years, age range: 8–92 years) were included in the analysis in Zhangqiu. The corresponding numbers of participants were 1022 (52.11 ± 13.07 years, age range: 8–80 years) and 1025 (51.63 ± 13.65 years, age range: 5–86 years) in Rushan. No significant differences were found in age and gender between HBsAg-positive and HBsAg-negative group in both counties (Zhangqiu: *P* = 0.232, 0.742; Rushan: *P* = 0.462, 0.471). In Zhangqiu, the HBsAg-positive group was consisted of 658 HBV carriers, 102 chronic hepatitis, 22 cirrhotic cases, 1 HCC case and 243 participants with unknown clinical type. In Rushan, the corresponding numbers were 838, 74, 8, 3 and 99.

**Table 1:** Demographic characteristics of HBsAg positive and negative participants in Zhangqiu and Rushan county, Shandong province, China

	Zhangqiu county			Rushan county		
	HBsAg-positive group (number, %)	HBsAg-negative group (number, %)	*P* value	HBsAg-positive group (number, %)	HBsAg-negative group (number, %)	*P* value
Total	1026 (100.00)	1029 (100.00)		1022 (100.00)	1025 (100.00)	
Age (yrs)						
Under 30	57 (5.56)	40 (3.89)	0.232	66 (6.46)	72 (7.02)	0.462
30–39	161(15.69)	170(16.52)		79(7.73)	79(7.71)	
40–49	284 (27.68)	275 (26.72)		242 (23.68)	237 (23.12)	
50–59	226 (22.03)	234 (22.74)		336 (32.88)	331 (32.29)	
60–69	196 (19.10)	200 (19.44)		211 (20.65)	222 (21.66)	
Above 70	102 (9.94)	110 (10.69)		88 (8.61)	84 (8.20)	
Gender						
Male	520 (50.68)	529 (51.41)	0.742	516 (50.49)	525 (51.22)	0.471
Female	506 (49.32)	500 (48.59)		506 (49.51)	500 (48.78)	

### Anti-HEV IgG seroprevalence among HBsAg-positive individuals

**Fig. 2:**
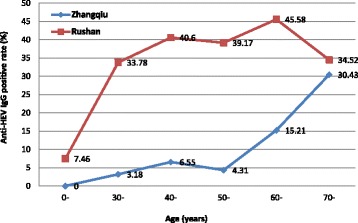
Anti-HEV IgG seroprevalence by age among HBsAg-positive participants in Zhangqiu county and Rushan county, Shandong province, China

HEV IgG seroprevalence was 9.16% (95% *CI* 7.47–11.09) in CHB group in Zhangqiu and was lower in comparison with that in Rushan (38.06%, *P* < 0.001). IgG seroprevalence increased with age in both counties (*P* < 0.01), although the seroprevalence was significantly higher in Rushan than in Zhangqiu in all age groups except those above 70 years (*P* < 0.05). IgG seroprevalnce was similar in males and females in Zhangqiu (*P* = 0.938), but was significantly different in Rushan (*P* < 0.001). The rate was much higher among HBV carriers in Rushan than in Zhangqiu (*P* < 0.001) and the same result was observed among hepatitis cases (*P* < 0.001). No significant difference in HEV IgG seroprevalence was found among different CHB groups in Zhangqiu (*P* = 0.714) and the same result was found in Rushan (*P* = 0.267). The details are shown in Tables 2, 3 and Fig. 2.

**Table 2:** Seroprevalence of hepatitis E IgG in HBsAg-positive group and HBsAg- negative group by age and gender in Zhangqiu and Rushan

	HBsAg-positive group		HBsAg-negative group		*P* value^#^*
	Number detected	Positive, *n* (%, 95% *CI*)	Number detected	Positive, *n* (%, 95% *CI*)	
Zhangqiu county					
Total	1026	94 (9.16,7.47–11.09)	1029	112 (10.88, 9.05–12.95)	0.21
Age (yrs)					
0-	53	0 (0.00, 0–6.72)	38	4 (10.53, 2.94–24.80)	0.03
30-	157	5 (3.18, 1.04–7.28)	167	7 (4.19, 1.70–8.44)	0.77
40-	275	18 (6.55, 3.92–10.15)	275	21 (7.64, 4.79–11.44)	0.74
50-	232	10 (4.31, 2.09–7.78)	237	24 (10.13, 6.60–14.69)	0.02
60-	217	33 (15.21, 10.70–20.69)	217	29 (13.36, 9.14–18.63)	0.68
70-	92	28 (30.43, 21.27–40.90)	95	27 (28.42, 1.96–38.60)	0.87
Gender					
Male	520	48 (9.23, 6.88–12.05)	500	52 (10.40, 7.86–13.41)	0.60
Female	506	46 (9.09, 6.73–11.94)	1029	112 (10.88, 9.05–12.95)	0.33
Rushan county					
Total	1022	389 (38.06, 35.07–41.19)	1025	407 (39.71, 36.70–42.78)	0.47
Age (yrs)					
0-	67	5 (7.46, 2.47–16.56)	73	15 (20.55, 11.98–31.62)	0.027
30-	74	25 (33.78, 23.19–45.18)	78	26 (33.33, 23.06–44.92)	1.00
40-	234	95 (40.60, 34.25–47.19)	234	94 (40.17, 33.84–46.76)	1.00
50-	337	132 (39.17, 33.92–44.60)	340	139 (40.88, 35.61–46.76)	0.70
60-	226	103 (45.58, 38.96–52.31)	225	100 (44.44, 37.84–51.20)	0.85
70-	84	29 (34.52, 24.48–45.69)	75	33 (44.00, 32.55–55.94)	0.26
Gender					
Male	516	216 (41.86, 37.56–46.25)	525	229 (43.62, 39.33–47.98)	0.57
Female	506	173 (34.19, 30.06–38.50)	500	178 (35.60, 31.40–39.97)	0.64

# Comparison between HBsAg positive and negative participants

*Fisher exact test

**Table 3:** Prevalence of anti-HEV IgG among HBsAg-positive participants by clinical types in Rushan county and Zhangqiu county, Shandong province, China

County	Clinical type	Number detected	HEV IgG(+) Number (%, 95% *CI*)	*P* value
Zhangqiu	HBV carrier	658	53 (8.05, 6.09–10.40)	0.714
	Chronic case	102	9 (8.82, 4.11–16.09)	
	Cirrhosis case	22	0 (0.00, 0–15.44)	
	HCC case	1	0 (0.00, 0–97.5)	
Rushan	HBV carrier	838	314 (37.47, 34.18–40.85)	0.267
	Chronic case	74	30 (40.54, 29.27–52.59)	
	Cirrhosis case	8	3 (37.50, 8.52–75.51)	
	HCC case	3	3 (100.00, 29.24, 100.00)	

*HBV* hepatitis B virus, *HCC* hepatocellular carcinoma

In the CHB group, the positive rate of HBeAg among anti-HEV IgG (+) participants was significantly lower than the rate among anti-HEV IgG (−) participants (17.18% vs 24.54%, *P* = 0.001), and the same trend was found in the proportion of participants with HBV DNA > 10^3^ copies/ml (32.92% vs 40.38%, *P* = 0.011). No significant difference was found between the participants positive for anti-HEV IgG and negative for anti-HEV IgG in the proportions of participants with elevated ALT level and the liver damage identified by ultrasound (*P* > 0.05). The details are shown in Table 4.

**Table 4:** The results of medical examination in HBsAg-positive participants by anti-HEV IgG

	Anti-HEV IgG positive		Anti-HEV IgG negative		*P* value
	Number	%	Number	%	
Total	483	100.00	1565	100.00	
HBeAg					
Positive	83	17.18	384	24.54	0.001
Negative	400	82.82	1181	75.46	
Anti-HBe					
Positive	313	64.80	1022	65.30	0.840
Negative	170	35.20	543	34.70	
HBV DNA (IU/ml)					
< 10^3^	324	67.08	933	59.62	0.011
10^3^–10^6^	97	20.08	404	25.81	
≥ 10^6^	62	12.84	228	14.57	
ALT(U/L)					
Normal	440	91.10	1442	92.14	0.463
Elevated	43	8.90	123	7.86	
Liver abnormality^a^					
Yes	5	1.04	13	0.83	0.674
No	478	98.96	1552	99.17	

^a^Identified by B ultrasound

### Anti-HEV IgM seroprevalence among HBsAg-positive individuals

Only one participant was positive for anti-HEV IgM in CHB group in Zhangqiu and the positive rate was 0.1% (95%*CI:* 0.002–0.54). The corresponding rate was 1.57% (16/1022, 95% *CI*: 0.90–2.53) in Rushan, which was much higher than the rate in Zhangqiu (*P* < 0.001). The positive rate of IgM in Rushan was not statistically significant by age, gender and clinical types (*P* > 0.05). HEV RNA has been tested for all serum positive for anti-HEV IgM, but none was positive. The details are shown in Table 5.

**Table 5:** Seroprevalence of anti-HEV IgM among population with chronic hepatitis B infection in Rushan county, Shandong province, China

	Number detected	Positive number	Positive rate (%, 95% *CI*)	*P* value
Total	1022	16	1.57 (0.90–2.53)	–
Age group (yrs)				
Under 30	66	0	0 (0–5.44)	0.768
30–39	79	1	1.26 (0.03–6.85)	
40–49	242	6	2.48 (0.92–5.32)	
50–59	336	5	1.49 (0.48–3.44)	
60–69	211	3	1.42 (0.29–4.10)	
Above 70	88	1	1.14 (0.03–6.17)	
Gender				
Male	516	8	1.55 (0.67–3.03)	0.969
Female	506	8	1.58 (0.68–3.09)	
Clinical type^a^				
HBV carrier	838	12	1.43 (0.74–2.49)	0.060^#^
Chronic cases	74	0	0 (0–4.86)	
Cirrhosis	8	0	0 (0–36.94)	
HCC	3	1	33.33 (0.84–90.57)	

*HBV* hepatitis B virus, *HCC* hepatic cellular cancer

# Fisher’s exact test

^a^Ninety nine participants with unknown clinical type were not involved in the analysis

### Comparison of HEV seroprevalence between CHB group and control group

HEV IgG seroprevalence was 9.16% and 10.88% in the CHB group and the control group, respectively, in Zhangqiu and the corresponding rates were 38.06% and 39.71% in Rushan. No significant difference between the two groups was found in either county (*P* = 0.21, *P* = 0.47, respectively). Age-specific and sex-specific seroprevalence of HEV IgG was similar between the CHB group and the control group except in age-groups of 0–29 years and 50–59 years in Zhangqiu and 0–29 years in Rushan. The positive rate of anti-HEV IgM was 0.1% and 0.48% respectively among CHB group and the control group in Zhangqiu (*P* = 0.103). The corresponding rates in Rushan were 1.57% and 2.24% and the difference was not significant too (*P* = 0.262).

Multivariable analysis showed that only age was independently associated with anti-HEV IgG in Zhangqiu (*P* < 0.05), and gender, age and education attainment were independently associated with anti-HEV IgG in Rushan (*P* < 0.05). The status of HBsAg was not independently associated with the status of anti-HEV IgG in either county (*P* > 0.05). The details are shown in Table 6.

**Table 6:** Multivariable analysis of the risk factors associated with anti-HEV IgG in Zhangqiu county and Rushan county, Shandong province, China

	Zhangqiu			Rushan		
	Participant number	*P*	*OR* (95% CI)	Participant number	*P*	*OR* (95% CI)
Gender						
Male	1049	Ref.	Ref.	1041	Ref.	Ref.
Female	1006	0.05	0.72 (0.52,0.98)	1006	< 0.001	0.71 (0.58,0.86)
Age (yrs)						
Under 30	97	Ref.	Ref.	138	Ref.	Ref.
30–39	331	0.54	0.71 (0.24,2.10)	158	< 0.001	3.38 (1.87,6.11)
40–49	559	0.46	1.45 (0.54,3.88)	479	< 0.001	4.64 (2.74,7.86)
50–59	460	0.41	1.53 (0.56,4.17)	667	< 0.001	4.17 (2.49,6.99)
60–69	396	0.02	3.22 (1.18,8.73)	433	< 0.001	5.47 (3.20,9.35)
Above 70	212	< 0.001	8.62 (3.08,24.15)	172	< 0.001	4.49 (2.45,8.22)
Education attainment						
Illiteracy	227	Ref.	Ref.	115	Ref.	Ref.
Primary school	739	0.246	1.32 (0.83,2.09)	560	0.025	1.66 (1.06,2.60)
Junior middle school	972	0.676	1.12 (0.64,1.96)	1121	0.037	1.63 (1.03,2.58)
Senior middle school and above	117	0.715	1.18 (0.48,2.92)	251	0.033	1.76 (1.04,2.96)
Occupation about swine laughter or selling						
Yes	11	Ref.	Ref.	61	Ref.	Ref.
No	2044	0.122	0.29 (0.06,1.39)	1986	0.654	1.13 (0.66,1.93)
Occupation about seafood cultivation, processing or selling						
Yes	0	–	–	43	Ref.	Ref.
No	2055	–	–	2004	0.103	0.59 (0.31,1.11)
Wash hands before dining						
≧2 times per day	2015	Ref.	Ref.	1877	Ref.	Ref.
≦one time per day	40	0.506	0.61 (0.14,2.63)	170	0.984	1.00 (0.72,1.39)
Drinking unboiled water						
≧3 times per week	51	Ref.	Ref.	143	Ref.	Ref.
1–2 times per week	417	0.637	1.32 (0.42, 4.12)	214	0.12	0.69 (0.44,1.09)
Less than once per week	1587	0.673	1.27 (0.42, 3.87)	1690	0.6	0.91 (0.63,1.31)
Eating outside						
≧3 times per week	94	Ref.	Ref.	98	Ref.	Ref.
1–2 times per week	611	0.926	1.04 (0.47,2.29)	140	0.924	0.97 (0.55,1.72)
Less than once per week	1350	0.644	0.83 (0.38,1.81)	1809	0.34	1.25 (0.79,1.96)
Chronic hepatitis B infection						
Yes	1026	Ref.	Ref.	1022	Ref.	Ref.
No	1029	0.187	1.23 (0.90,1.68)	1025	0.609	1.05 (0.87,1.26)

## Discussion

Our study showed similar HEV seroprevalence between HBsAg-positive and negative participants in areas with different HEV endemicity. This result is consistent with some previous studies [13, 23], but different from the study by Hoan NX [15]. The reason for the above difference might be due to differences in the participants in these studies. Our study is community-based and most HBsAg-positive participants are HBV carriers, while Hoan NX’s study is hospital-based and most participants are chronic hepatitis patients. HEV genotype 4 is predominant in Shandong province [24, 25], and its epidemiology differs from other genotyopes [5, 26], so our finding should be further studied in other areas with different HEV genotypes.

Our study has provided the preliminary evidence for high incidence of HBV-HEV superinfection, in HEV hyper-endemic areas. According to our study, positive results for anti-HEV IgG were found approximately among 40% of HBsAg-positive persons in Rushan. In China, most chronic HBV infections occur at birth or early childhood, while most HEV infections occur in adults [18]. So it is most likely that HEV infection in our study participants had occurred after HBV infection. Although the duration of the persistence of anti-HEV IgG is still unclear, it is sure not to be life-long [27, 28]. Given this fact, the real seroprevalence of HEV among HBsAg-positive participants might be higher than what we observed in the study.

The detection of anti-HEV IgM could give the direct evidence of acute hepatitis E infection among persons with chronic HBV infection. Although the positive rate of anti-HEV IgM was very low among HBsAg-positive participants in Rushan in our study (only 1%), it suggests a high risk to attack HEV acute infection among HBsAg-positive patients in the county because this result came from a cross-section study instead of a cohort study.

Some studies showed that HEV infection could result in severe disease in patients with underlying CHB even liver failure [8, 29–31], but the other obtained the opposite conclusion [32]. We did not find more active HBV replication and more severe live damage in HBsAg(+), anti-HEV IgG(+) participants than in HBsAg(+), anti-HEV IgG(−) participants in the present study. However, it must be noted that most participants were HBV carriers in our study, while most previous studies were conducted among patient with chronic hepatitis or in cirrhotic patients.

Hepatitis E vaccine was licensed in China in 2011 and is the unique commercially available hepatitis E vaccine in the world till now [33]. The immunogenicity and safety of hepatitis E vaccine has been documented in HBV carriers [34], but no similar studies are available among CHB, cirrhosis and HCC patients. Given the fact that most CHB patients develop from HBV carriers and the long-lasting anti-HEV IgG induced by hepatitis E vaccine has been documented [35], hepatitis E vaccine is recommended to HBV carriers to prevent HBV-HEV superinfection, especially in HEV hyperendemic areas.

Two counties with different HEV prevalent level were included in the study, giving the study more depth and improved the generalisability of the findings. However, due to the community-based design of the study, most HBsAg-positive participants were carriers in our study and the conclusions should be further documented in clinical cases.

## Conclusions

The HEV seroprevalence is similar between HBsAg-positive and HBsAg-negative participants in the same area. The risk of HBV-HEV superinfection could vary greatly in different areas and the HBsAg-positive persons living in HEV hyperendemic areas might be at higher risk for HBV-HEV superinfection. Further study should be conducted to determine the epidemiological characteristics and clinical outcome of HEV-HBV superinfection in HEV hyperendemic areas.

## Abbreviations

Anti-HBs: antibody against HBsAg
CHB: Chronic hepatitis B
CI: Confidential interval
HBsAg: Hepatitis B surface antigen
HBV: Hepatitis B virus
HCC: Hepatocellular carcinoma
HEV: Hepatitis E virus
